# Alternative application of Tau protein in Creutzfeldt-Jakob disease diagnosis: Improvement for weakly positive 14-3-3 protein in the laboratory

**DOI:** 10.1038/srep15283

**Published:** 2015-10-28

**Authors:** Jae Wook Hyeon, Su Yeon Kim, Jeongmin Lee, Jun Sun Park, Kyu Jam Hwang, Sol Moe Lee, SeongSoo A. An, Myung Koo Lee, Young Ran Ju

**Affiliations:** 1 Division of Zoonoses, Center for Immunology & Pathology, National Institute of Health, Korea Centers for Disease Control and Prevention, Chungcheongbuk-do 28159, Korea; 2 Gachon BioNano Research Institute, Gachon University, Gyeonggi-do 461-701, Korea; 3 College of Pharmacy, Chungbuk National University, Cheongju 361-763, Korea

## Abstract

The 14-3-3 protein has been used as a biomarker for the diagnosis of sporadic Creutzfeldt-Jakob disease (sCJD). However, weakly positive 14-3-3 leads to false positive results and an incorrect diagnosis. We attempted to use quantitative data for tau protein to provide an accurate diagnosis based on weak 14-3-3 protein. Sixty-two patients with sCJD, including pathologically confirmed, clinically definite, and probable cases, and 89 non-CJD patients were investigated based on a Korean population. Among them, 20 sCJD and 14 non-CJD showed weakly positive 14-3-3. The total tau (t-tau) and phosphorylated tau (p-tau) protein levels were measured by ELISA, and the p-tau to t-tau ratio (p/t ratio) was calculated. The combined use of the 14-3-3 protein assay, t-tau levels, and p/t ratio improved the specificity of diagnosis compared with the use of the 14-3-3 protein assay alone (47% for 14-3-3 alone; 85.94% for 14-3-3 combined with t-tau; 90.62% for 14-3-3 combined with the p/t ratio). In addition, 18 of 20 sCJD and 12 of 14 non-CJD who were weakly positive for 14-3-3 were positive for the p/t ratio and negative for the p/t ratio, respectively. When used in combination with the 14-3-3 protein, the tau protein is useful as a biomarker for the precise diagnosis of sCJD.

Prion diseases are fatal neurodegenerative diseases that affect both humans and animals. In humans, the etiology of these diseases is unknown. However, the conformational conversion of the cellular prion protein PrP^C^ into PrP^Sc^, a proteinase K-resistant misfolded isoform, is thought to be the cause of these diseases^1^. A definite diagnosis of prion diseases, such as sporadic Creutzfeldt–Jakob disease (sCJD), is established based on the demonstration of the presence of PrP^Sc^ in the brain tissue. A diagnosis of probable or possible sCJD is established based on clinical features, periodic sharp and slow waves on electroencephalography (EEG), brain magnetic resonance imaging (MRI), and the presence of the 14-3-3 protein in the cerebrospinal fluid (CSF)^2^. The 14-3-3 proteins are a group of cytosolic polypeptides that are released into the CSF in disease states such as CJD, stroke, infections, inflammatory diseases, epileptic seizures, and toxic metabolic conditions^3^,^4^.

Determination of the presence of the 14-3-3 protein in the CSF is complicated by the variability of factors such as the experimental methods, antibody epitopes, exposure times, and enhanced chemiluminescence (ECL) solutions used to develop the blots. Thus, the reported 14-3-3 protein sensitivities vary between 43% and 100%^5^,^6^,^7^,^8^,^9^, and the specificities vary between 47% and 97%^7^,^10^,^11^. However, the case surveillance definitions still include the 14-3-3 assay. CSF 14-3-3 protein detection is qualitative and somewhat subjective. Some researchers have tried to improve these aspects by developing a 14-3-3 enzyme-linked immunosorbent assay (ELISA)^9^,^12^. This method was demonstrated as a potential diagnostic tool but is not yet widely used for CJD diagnosis in the laboratory. The analytic cut-off data are considerably important and sensitive. The cut-off should be selected based on the normalized values using comparisons among many studies. In a similar manner, the CSF from sCJD patients and recombinant 14-3-3 protein are suitable as positive controls in western blots of the CSF 14-3-3 protein detection. The CSF from patients without sCJD or normal individuals should be used as a negative control. However, CSF specimens with an unknown status do not always show an obvious intensity compared with the controls. Such ambiguous intensity has often been called weakly positive. This result causes confusion to the clinicians regarding the final diagnosis.

In addition to the 14-3-3 protein, other biomarkers such as total tau (t-tau), phosphorylated tau (p-tau), astrocytic S100b, and neuron-specific enolase (NSE) have been reported as being useful for the discrimination of sCJD from other neurodegenerative diseases^7^,^10^,^11^. The tau protein has been reported to be an alternative biomarker for the diagnosis of patients with sCJD^10^,^11^,^13^,^14^,^15^. Our study was performed to improve the limit of 14-3-3 protein detection through a combination analysis with tau values (t-tau, p-tau, and p/t ratio) using the cut-off based on our cases. True or false positives for the 14-3-3 protein should be distinct from weakly positive.

Among these markers, the tau protein is found in neurofibrillary tangles (NFTs). Its malfunction leads to Alzheimer’s disease (AD) and other tauopathies^16^. The 14-3-3 protein is also found in NFTs. Studies have shown a relationship between the tau and 14-3-3 proteins. Otto *et al*. concluded that isoforms of 14-3-3 are involved in the action of p-tau under pathological conditions^10^. The presence of high levels of tau protein in the CSF of sCJD patients would be evidence and information that release of the protein is enhanced in CSF as well as tissue samples because of the neuronal dysfunction.

The definite diagnosis of sCJD requires a biopsy or autopsy of the brain tissue. However, these methods are not often acceptable or desirable in Korea because of the cultural background and the difficulty of the medical procedures, which reflect the low number of definite CJD cases reported in Korea. A number of groups have attempted to develop reliable and effective biomarkers for the antemortem diagnosis of sCJD, including the quantification of the tau protein in the CSF of sCJD patients^10^,^17^. Here, we examined the utility of the tau protein as an additional marker to improve the accuracy of sCJD diagnosis. To this end, we measured the t-tau and p-tau levels in the CSF. We report that a more accurate antemortem diagnosis of sCJD can be made by the combined analysis of t-tau, p-tau, and 14-3-3 present in the CSF.

## Results

### Tau protein assay

**Figure 1:**
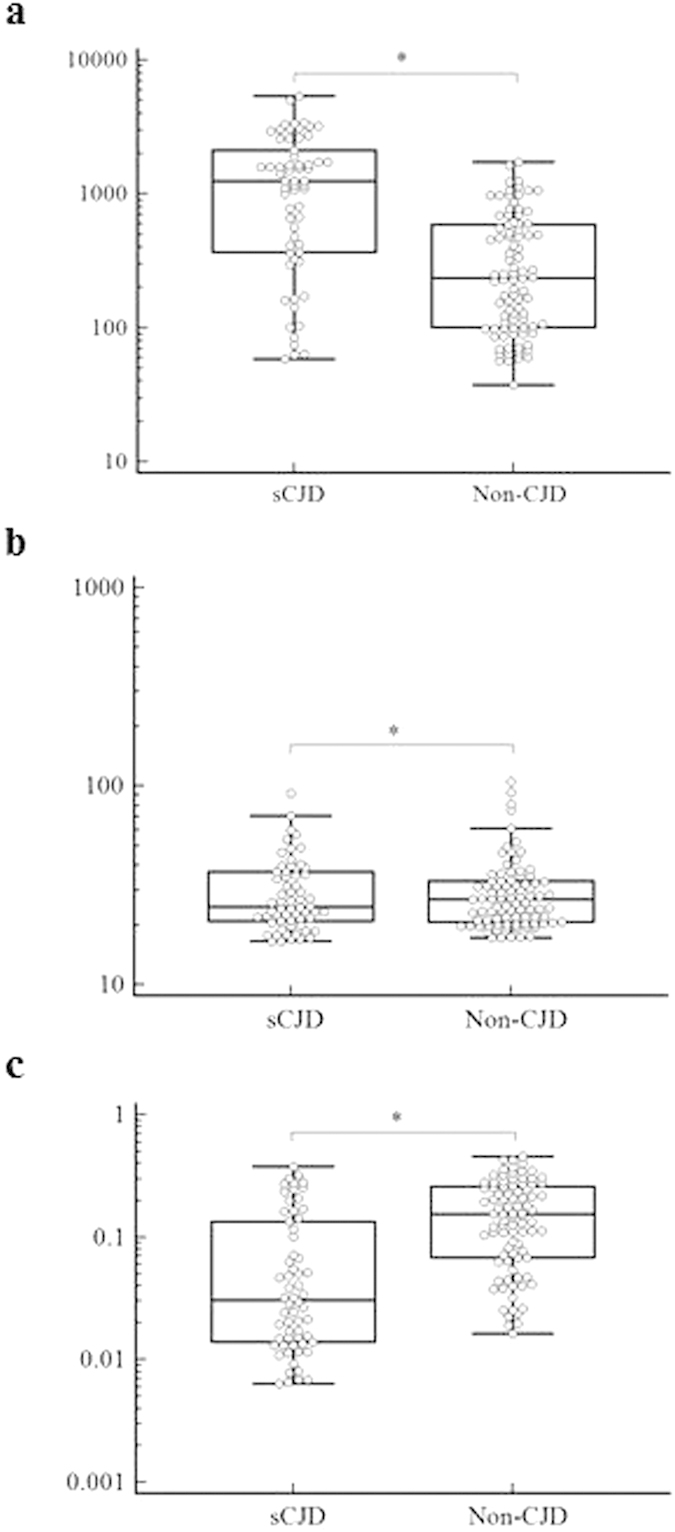
Box plot showing the CSF levels of (a) t-tau, (b) p-tau, and (c) p/t ratio in sCJD and non-CJD patients on a log scale (y-axis). The plots show the 10th, 25th, 50th, 75th, and 90th percentiles and the outliers. *Significance levels are *P* < 0.001.

The distributions of t-tau and p-tau and the p/t ratio in the sCJD and non-CJD groups were determined. The t-tau level and p/t ratio in the sCJD group significantly differed from those in the non-CJD group (*P* < 0.001). The t-tau level in the sCJD group was considerably higher than that in the non-CJD group (Fig. 1a). The p-tau level was similar in both groups (Fig. 1b). In the sCJD group, the p/t ratio was low (Fig. 1c).

The ROC curves were plotted for the t-tau level, p-tau level, and p/t ratio in the sCJD and non-CJD groups. The diagnostic sensitivity (Se) and specificity (Sp) were optimized by the Youden index J (Se + Sp − 1). While calculating the cut-off values for the sCJD and non-CJD groups, the best results were obtained at 1,069.9 pg/mL for t-tau, 18.9 pg/mL for p-tau, and 0.067 for the p/t ratio. The area under the ROC curve (AUC) estimated using the Hanley and McNeil method was 0.788 (95% CI 0.718–0.850, *P* < 0.0001) for t-tau, 0.514 (95% CI 0.432–0.963, *P* = 0.7687) for p-tau, and 0.764 (95% CI 0.689–0.830, *P* < 0.0001) for the p/t ratio. At this point, the sensitivity and specificity of t-tau were 59.68% (95% CI 46.4–71.9) and 94.38% (95% CI 87.4–98.2), respectively. The sensitivity and specificity of p-tau were 19.35% (95% CI 10.4–31.4) and 92.13% (84.5–96.8), respectively. The sensitivity and specificity of the p/t ratio were 69.35% (95% CI 56.3–80.4) and 76.40% (95% CI 66.2–84.8), respectively. The positive likelihood ratio (+LR) at our estimated cut-off values was the highest for t-tau at 10.62 (95% CI 4.4–25.5). The +LR values for p-tau and the p/t ratio were 2.46 (95% CI 1.0–5.9) and 2.94 (95% CI 2.0–4.4), respectively. The negative likelihood ratio (-LR) was the lowest for the p/t ratio at 0.40 (95% CI 0.3–0.6). The –LR values for t-tau and p-tau were 0.43 (95% CI 0.3–0.6) and 0.88 (95% CI 0.8–1.0), respectively (Table 1).

### 14-3-3 protein assay

**Figure 2:**
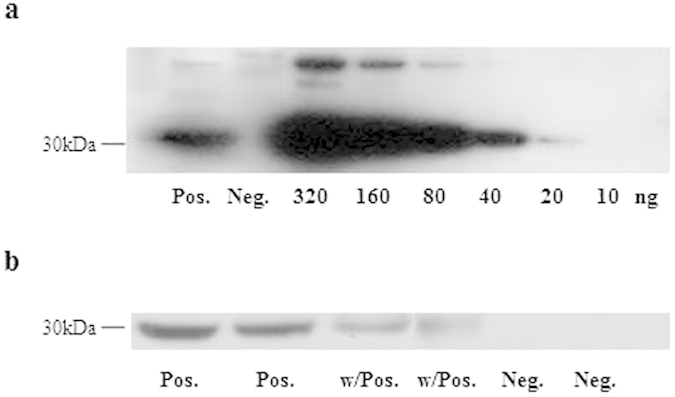
Western blot analysis of the CSF 14-3-3 protein. Serial dilutions of 10, 20, 40, 80, 160, and 320 ng of recombinant 14-3-3 protein were analyzed (**a**). Representative 14-3-3 positive, weakly positive and negative samples are displayed (**b**). Pos. indicates 14-3-3-positive in sCJD patients. Neg. indicates 14-3-3-negative in non-CJD patients. w/Pos. and w/Neg. indicate weakly positive and weakly negative, respectively.

Sixty-one cases (98.4%) were positive 14-3-3 protein. All pathologically confirmed and clinically definite cases were 14-3-3 positive (100%). Among the probable cases, 40 (97.5%) were 14-3-3 positive. The threshold for 14-3-3 positive corresponded to the immunoreactivity of approximately 40 ng of recombinant 14-3-3 protein. Weakly positive corresponded to the immunoreactivity of approximately 20 ng of recombinant 14-3-3 protein (Fig. 2). The distribution of the 14-3-3 protein was differentiated in both groups (Table 2). In the sCJD group, 61/62 cases were positive 14-3-3 protein, including 20 cases (approximately 30%) that were weakly positive. In the non-CJD group, 44/89 cases were positive 14-3-3 protein, including 14 weakly positive cases.

### Combined analysis for 14-3-3 and tau proteins

**Figure 3:**
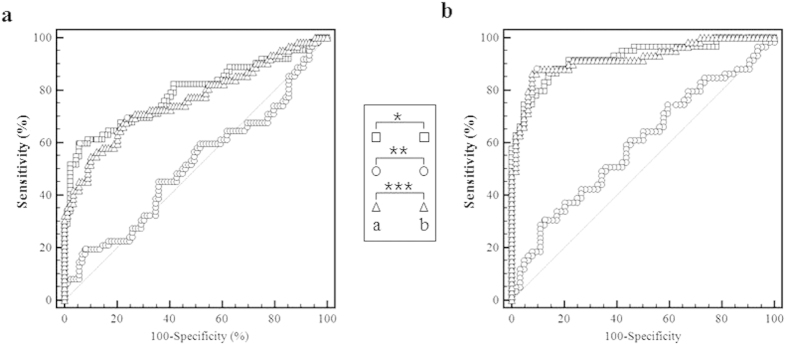
Receiver operating characteristic (ROC) curves plotted for a total of 151 patients. The single analyses for t-tau, p-tau, and p/t ratio are represented (**a**). The combined analyses for t-tau, p-tau, and p/t ratio together with the 14-3-3 protein are shown in (**b**). The square, circle, and triangle markers indicate the ROC curves for t-tau, p-tau, and p/t ratio, respectively. The small box represents the statistical comparison of t-tau, p-tau and p/t ratio in between (**a** and **b**). Significance levels are *P < 0.0001, **P = 0.6574, ***P < 0.0001. The dotted line indicates the diagonal representing a hypothetical test with no diagnostic discrimination.

Each case positive for t-tau, p-tau, p/t ratio, and 14-3-3 was considered to be a single positive case. The double positive and negative cases were plotted as groups of sCJD and non-CJD patients (Fig. 3a). The t-tau level and the p/t ratio appeared to be significantly promising markers (Table 3). These AUCs were considerably elevated compared with the single values, which were 0.922 (95% CI 0.860–0.963, *P* < 0.0001) for t-tau and 0.922 (95% CI 0.860–0.963, *P* < 0.0001) for the p/t ratio. The sensitivity was also elevated in the combined analysis: 86.44% (95% CI 75.0–94.0) for t-tau and 88.14% (95% CI 77.1–95.1) for the p/t ratio. The specificity of the p/t ratio was elevated at 90.62% (95% CI 80.7–96.5) (Fig. 3b). By contrast, the specificity of t-tau was reduced at 85.94 (95% CI 75.0–93.4). The +LR was the highest for the p/t ratio at 9.40 (95% CI 4.4–20.3). The +LR of t-tau was 6.15 (95% CI 3.3–11.4). The −LR was the lowest for the p/t ratio at 0.13 (95% CI 0.07–0.3). The −LR of t-tau was 0.16 (95% CI 0.08–0.3).

### Weakly positive 14-3-3 protein with tau values

Applying the t-tau and p/t ratio levels, we evaluated the 34 weakly positive 14-3-3 cases, which could be either true positives or false positives (Table 4). The weakly positive 14-3-3 cases were distributed across the sCJD and non-CJD groups. In the sCJDgroup, 7 of 20 cases were positive for t-tau. Remarkably, 18 of 20 cases were positive for the p/t ratio. Of the 14 cases in the non-CJD group, 14 were negative for t-tau, 12 were negative for the p/t ratio, and one was negative for p-tau. The p-tau value was positive in almost all (31 of 34) weakly positive 14-3-3 cases. The analysis of the tau values combined with weakly positive 14-3-3 protein indicated that the p/t ratio was the most effective marker for discriminating among patients in both groups.

## Discussion

Here, we report the sole or joint performance of multiple markers, including the 14-3-3 protein, t-tau and p-tau levels and the p/t ratio, in accurately discriminating between sCJD patients, including pathologically confirmed, clinically definite, and probable cases, and non-CJD patients. Our data, as well as those of others, also showed elevated t-tau and nearly unchanged p-tau levels in patients with sCJD^15^,^18^,^19^. Therefore, the p/t ratio is likely to depend on the t-tau levels. As seen in Fig. 1, p-tau was concentrated in the 25th–75th percentiles in both groups. The diagnostic value of p-tau alone was poor. However, this value is needed to calculate the p/t ratio. Our findings suggest that because of the greater variation in CSF, the t-tau levels affect the p/t ratio to a greater extent than p-tau. Although the methods used for assaying 14-3-3 vary widely, the tau assays are mostly performed using a single tau ELISA kit manufactured by Innogenetics. We measured the optimal decision point using the ROC curve in our selected population. These points were different from what has been reported. Previous reports suggested that only the measurement of t-tau had a high impact on the diagnosis of sCJD, reaching a sensitivity and specificity of approximately 90%, which fell within a reliable range^20^,^21^.

It was verified that the combination of the p/t ratio and 14-3-3 protein enabled a more accurate diagnosis than either the 14-3-3 or tau values alone. The usefulness of the p-tau protein in the diagnosis of sCJD is important in the calculation of the p/t ratio. There were many negative cases for t-tau in the non-CJD group, showing its potential as an adequate marker for the diagnosis of sCJD. The antibody epitopes for t-tau and p-tau in the ELISA by Innogenetics do not overlap, and p-tau and normal tau (non-phosphorylated) were both detected in t-tau, whereas p-tau was specific at the thr181 position. Therefore, we consider that the criteria should preferably include the relative level of p-tau among t-tau. We determined that the sensitivity was increased when the p/t ratio value was applied and that both sensitivity and specificity were increased with the addition of the 14-3-3 results. It was verified that we could obtain an optimal accuracy using the combination of the 14-3-3 value with the t-tau, p-tau and p/t ratio values.

Weakly positive 14-3-3 protein may lead to an incorrect or inconclusive diagnosis of sCJD. As seen in Table 4, those cases that are positive for the p/t ratio and weakly positive for 14-3-3 may be closer to sCJD than the cases that are weakly positive for 14-3-3 only. Additionally, those cases that are negative for t-tau and the p/t ratio and weakly positive for 14-3-3 may be closer to non-CJD. Our results demonstrate that t-tau and the p/t ratio can be used to reinforce the 14-3-3 positive-based diagnostic method and to obtain a more accurate diagnosis of sCJD.

The clinical application of the 14-3-3 protein remains controversial, with high sensitivity and low specificity resulting in false positives or negatives^6^,^7^. Although the precise reason for this inconsistency is unclear at present, the heterogeneity of sCJD appears to influence the diagnostic results^22^. We observed atypical background patterns on an immunoblot when the results of the 14-3-3 test were all positive. This observation may represent meaningful evidence for the presence of unknown subtypes or patterns in sCJD.

Another potential explanation for the inconsistencies encountered is the differences in the sampling methods and SOPs used. Interestingly, Sanchez-Valle *et al*. showed that regardless of the temperature, the 14-3-3 protein is very stable in the CSF^23^. Unlike in other countries, CJD surveillance by the KCDC is not under the immediate control of a medical institution. Once a lumbar puncture is performed, the samples are transported over the course of a few days at 4 °C. Upon arrival in the laboratory, the samples are stored at −80 °C until use. The long-term storage after CSF collection and temperature changes before testing may explain the inconsistencies in the results. CSF samples have often been found to be contaminated with blood. Careless CSF sample collection via lumbar puncture would thus yield misleading results^14^. In the present study, the presence of blood was detected in nine samples, and all nine cases were positive for 14-3-3.

The SOPs have not been standardized worldwide. Most laboratories have their own SOPs for the 14-3-3 assay, which results in significant methodological differences. The determination of the 14-3-3 protein level in the CSF depends on antibody epitopes, transfer methods, exposure times, and ECL solutions. Most importantly, the antibodies against the 14-3-3 protein isotype are not standardized for diagnostic use. The appearance of the bands on the immunoblot is determined by the isotype, monoclonal/polyclonal nature, working concentration, and manufacturer (laboratory or company) of the antibody. Furthermore, the variations in the numbers of sCJD and non-CJD group members affect the dependability of the analysis results. A number of studies have collected data from a broad range of patients with suspected CJD^6^,^7^,^14^,^22^,^24^. Therefore, statistically, the sensitivity and specificity of the test have varied. Our major results were based on a Korean population

Finally, the 14-3-3 immunoblot is performed in a qualitative manner, and conclusions are derived based on subjective visual interpretations. A standardized approach to the analysis of the bands, including the decision to classify weakly positive or ambiguous bands as being positive results versus classifying only intense bands as positive results for the 14-3-3 protein, is lacking. To eliminate the differences originating from the subjective interpretation of the results, the use of a densitometric and quantitative analysis of the 14-3-3 protein has been proposed^6^. 14-3-3 proteins interact with more than 200 proteins involved in control of the cell cycle, gene expression, and apoptosis, through phospho-dependent or-independent interactions^25^,^26^,^27^. It has been reported that 14-3-3 proteins are detected in NFTs. This suggests the possibility that the phosphorylation of tau may be linked to the presence of 14-3-3 proteins^28^,^29^. Furthermore, 14-3-3 *β*, *η*, and *ζ* forms have been shown to bind with high affinity to tau that has been phosphorylated by protein kinase A (PKA), protein kinase B (PKB), and glycogen synthase kinase-3beta (GSK3*β*)^30^,^31^. We believe that the 14-3-3 proteins clearly affected the phosphorylation of tau in sCJD, that the markers is pathologically increased in AD, and that 14-3-3 *β* may also interact with p-tau, although this remains to be determined by cellular localization studies performed with specific 14-3-3 isoforms and other binding proteins associated with neurodegenerative disorders.

In summary, our results show that compared with the use of a single marker, the use of a combination of 14-3-3 positivity and the p/t ratio significantly improved the specificity of the diagnostic test to an optimal level. As described earlier, the biopsy and autopsy of brain tissues of sCJD patients are essential for the diagnosis of definite sCJD. However, within oriental cultures, many patients or their caretakers frequently reject these invasive procedures. An antemortem diagnosis is only required to minimize the biopsy and autopsy. In addition to the 14-3-3 protein, a second biomarker should be introduced to enhance the accuracy of the sCJD diagnosis. Such a marker will minimize the dependence on the definitive procedure. Therefore, tau, a second marker protein in the CSF, combined with the 14-3-3 protein could be used in clinical practice for the antemortem diagnosis of sCJD.

## Methods

### Subjects and their characteristics

All suspected CJD patients showing symptoms of neurological disorders, including cognitive decline or motor impairment, were asked to undergo CJD diagnosis from the years 2005 to 2012. Sixty-two sCJD patients who fulfilled the criteria and 89 non-CJD samples with an inconclusive diagnosis were selected for this study. The diagnosis of sCJD was made according to the revised WHO criteria with the help of MRI findings. Patients with a neuropathological diagnosis, including the immunohistochemical detection of PrP^Sc^ in the brain tissue, were considered “pathologically confirmed.” Patients with rapidly progressive dementia of <2 years’ duration, myoclonus, and appropriate EEG and MRI findings, but no biopsy or autopsy results, were considered “clinically definite.” A diagnosis of “probable” was assigned to patients with progressive dementia, myoclonus, EEG or MRI findings or the presence of 14-3-3 in the CSF. The “non-CJD group” was defined as not fulfilling the criteria for possible or probable sCJD but having an inconclusive diagnosis for sCJD. The patient profiles, including sex, disease onset, 14-3-3 test results, and genotyping results, are shown in Table 5. Sixty-two sCJD patients were classified as follows: 5 “pathologically confirmed” cases, 16 “clinically definite” cases, and 41 “probable” cases. Of the 62 sCJD cases, 36 were males (58%), and 26 were females (42%); 56 (90.3%) were older than 50 years of age (Table 5). Of the 89 non-CJD cases, 51 (57.3%) were males, and 38 (42.7%) were females; additionally, 79 (88.8%) were older than 50 years of age. The *PRNP* codon 129 genotypes among the sCJD cases included 60 (96.7%) M/M, 2 (3.3%) M/V, and 0 (0%) V/V. In the non-CJD group, there were 86 (96.6%) M/M, 3 (3.4%) M/V, and 0 (0%) V/V. They had been examined for a CJD diagnosis based on symptoms of depression, lack of memory, some ataxia and dementia, but they were not clinically classified as having other diseases.

### Genotyping the *PRNP* gene

Genetic analysis of the prion protein gene (*PRNP*) mutation and that of the polymorphism at codon 129 were performed using DNA extracted from blood specimens according to standard methods^32^.

### Immunoblotting of the CSF 14-3-3 protein

The immunoassay of the 14-3-3 protein was performed using our standard operating procedure (SOP), which was approved by the institutional committee of the KCDC. Briefly, the CSF proteins were separated on 4–12% SDS-PAGE at 180 V (50 min). The separated proteins were transferred to a PVDF membrane using a dry blot system (Invitrogen CA, USA). The membranes were blocked with 5% skim milk and incubated with human 14-3-3β antibody (Santa Cruz Biotechnology sc-629, CA, USA). Next, the membranes were incubated with horseradish peroxidase (HRP)-conjugated anti-rabbit IgG. The specific immunocomplexes were visualized with ECL detection reagents and imaged on film (Eastman Kodak Company, Rochester, NY, USA). The 293T cell lysate (Santa Cruz Biotechnology sc-111573, CA, USA) and a 14-3-3-positive CSF sample from a definite sCJD patient were used as positive controls. A 14-3-3-negative CSF sample from a non-CJD patient was used as a negative control. Two analytical scientists independently determined the 14-3-3 protein positive status by a visual inspection of the bands on the western blots according to the SOP.

### ELISA of the CSF tau protein

The t-tau and p-tau proteins were assayed using the Innotest^®^ hTau Ag and Innotest^®^ Phospho-Tau_(181P)_ ELISA kits (Innogenetics, Ghent, Belgium)) according to the manufacturer’s instructions. The absorbance at 450 nm was measured using a plate reader (VICTOR3, PerkinElmer, MA, USA). All samples were analyzed in duplicate, and the p-tau to t-tau ratio (p/t ratio) was calculated.

### Statistical analysis

MedCalc^®^ version 13.0.6.0 (MedCalc software, Mariakerke, Belgium) was used for the statistical analyses and preparing the graphs. The statistical analysis of the t-tau and p-tau levels and the p/t ratio between the sCJD and non-CJD groups was performed using the chi-square test. The receiver operating characteristic (ROC) analysis was performed using the method of Hanley and McNeil. A sample positive for both the tau value and 14-3-3 content was regarded as a single tau and 14-3-3 protein double-positive case. Similarly, a sample negative for both the tau value and 14-3-3 content was regarded as a single tau and 14-3-3 protein double-negative case.

### Ethics statement

The institutional committee of the Korea Centers for Disease Control & Prevention (KCDC) approved the “Western blot assay for 14-3-3 protein detection of sCJD diagnosis in the laboratory (KCDC-ZOO-CJD-WB-QIS-10-01)” as a standard protocol. The methods using human biological resources were performed in accordance with the guideline of the Institutional Review Board (IRB) of the KCDC. All patients enrolled in this study provided their informed consent.

## Additional Information

**How to cite this article**: Hyeon, J. W. *et al*. Alternative application of Tau protein in Creutzfeldt-Jakob disease diagnosis: Improvement for weakly positive 14-3-3 protein in the laboratory. *Sci. Rep.* **5**, 15283; doi: 10.1038/srep15283 (2015).

## Figures and Tables

**Table 1:** Statistical analysis of the receiver operating characteristic (ROC) curves for t-tau, p-tau, and p/t ratio.

Marker	Criterion^a^	Se^b^ (95% Cl)	Sp^b^ (95% Cl)	+LR^b^ (95% Cl)	−LR^b^ (95% Cl)	Significance levels	AUC^c^
t-tau	>1069.9	59.68 (46.4–71.9)	94.38 (87.4–98.2)	10.62 (4.4–25.5)	0.43 (0.3–0.6)	P < 0.0001	0.788 (0.718–0.850)
p-tau	>18.9	19.35 (10.4–31.4)	92.13 (84.5–96.8)	2.46 (1.0–5.9)	0.88 (0.8–1.0)	P = 0.7687	0.514 (0.432–0.596)
p/t ratio	≤0.067	69.35 (56.3–80.4)	76.40 (66.2–84.8)	2.94 (2.0–4.4)	0.40 (0.3–0.6)	P < 0.0001	0.764 (0.689–0.830)

^a^Optimal scoring thresholds for t-tau, p-tau and p/t ratio based on Youden Index J, estimated in this study. Values are in pg/ml for t-tau and p-tau.

^b^Se: sensitivity, Sp: specificity, +LR: positive likelihood ratio, −LR: negative likelihood ratio.

^c^AUC: area under the ROC curve.

**Table 2:** Overall diagnostic decision for single CSF markers in the sCJD and non-CJD groups.

CSF markers	sCJD (62)			Non-CJD (89)		
	Positive	Negative	Weakly positive	Positive	Negative	Weakly positive
14-3-3	41	1	20	30	45	14
t- tau	33	29	-	9	80	-
p- tau	50	12	-	82	7	-
p/t ratio	42	20	-	24	65	-

**Table 3:** Statistical analysis of the receiver operating characteristic (ROC) curves for t-tau, p-tau, and p/t ratio together with the 14-3-3 protein.

Marker*	Se^a^ (95% Cl)	Sp^a^ (95% Cl)	+LR^a^ (95% Cl)	-LR^a^ (95% Cl)	Significance level	AUC^b^
t-tau w/14-3-3+	86.44 (75.0–94.0)	85.94 (75.0–93.4)	6.15 (3.3–11.4)	0.16 (0.08–0.3)	P < 0.0001	0.922 (0.860–0.963)
p-tau w/14-3-3	30.51 (19.2–43.9)	87.50 (76.8–94.4)	2.44 (1.1–5.2)	0.79 (0.7–1.0)	P = 0.0667	0.594 (0.502–0.682)
p/t ratio w/14-3-3	88.14 (77.1–95.1)	90.62 (80.7–96.5)	9.40 (4.4–20.3)	0.13 0.07–0.3)	P < 0.0001	0.922 (0.860–0.963)

^*^Each joint marker was estimated by a combination of individual positive and negative results.

^+^Positive in both t-tau value and 14-3-3 measurement was regarded as one positive case of t-tau with the 14-3-3 protein.

^a^Se: sensitivity, Sp: specificity, +LR: positive likelihood ratio, -LR: negative likelihood ratio,

^b^AUC: area under the ROC curve.

**Table 4:** Application of the t-tau, p-tau, and p/t ratio to the 34 weakly positive 14-3-3 cases.

Case No	t-tau (pg/ml)	t-tau decision	p-tau (pg/ml)	p-tau decision	p/t ratio	p/t ratio decision	Groups
1	1109.80	P	35.62	P	0.032	P	sCJD
2	999.99	N	24.19	P	0.024	P	
3	1498.40	P	20.82	P	0.014	P	
4	1641.60	P	34.81	P	0.021	P	
5	1061.21	N	49.68	P	0.047	P	
6	739.60	N	27.83	P	0.038	P	
7	312.05	N	19.65	P	0.063	P	
8	210.04	N	24.36	P	0.116	N	
9	159.27	N	22.60	P	0.142	N	
10	814.58	N	26.36	P	0.032	P	
11	665.92	N	31.41	P	0.047	P	
12	674.30	N	26.33	P	0.039	P	
13	2627.07	P	16.63	N	0.006	P	
14	3037.12	P	39.74	P	0.013	P	
15	775.10	N	20.59	P	0.026	P	
16	1596.10	P	21.66	P	0.014	P	
17	1594.70	P	24.18	P	0.015	P	
18	995.30	N	31.99	P	0.032	P	
19	365.40	N	18.90	N	0.052	P	
20	699.10	N	28.95	P	0.041	P	
21	85.32	N	23.39	P	0.274	N	Non-CJD
22	106.18	N	36.38	P	0.343	N	
23	90.43	N	27.42	P	0.303	N	
24	98.18	N	27.35	P	0.279	N	
25	159.95	N	40.29	P	0.252	N	
26	69.64	N	17.15	N	0.246	N	
27	146.68	N	32.88	P	0.224	N	
28	135.65	N	23.47	P	0.173	N	
29	256.16	N	32.17	P	0.126	N	
30	222.25	N	24.78	P	0.112	N	
31	177.00	N	19.56	P	0.111	N	
32	410.97	N	22.03	P	0.054	P	
33	592.12	N	25.93	P	0.044	P	
34	71.02	N	26.87	P	0.378	N	

**Table 5:** Demographics of the sCJD and non-CJD groups.

Items & Profiles, N (%)	sCJD, 62	Three categories of sCJD, 62			Non-CJD, 89
		Pathologically confirmed, 5	Clinically definite, 16	Probable, 41	
Male	36 (58.0)	0 (0)	12 (75.0)	21 (51.2)	51 (57.3)
Female	26 (42.0)	2 (40.0)	4 (25.0)	20 (48.8)	38 (42.7)
Age at onset <50	6 (9.7)	1 (20.0)	1 (6.3)	4 (10.0)	10 (11.2)
Age at onset <50–70	36 (58.1)	3 (60.0)	7 (43.7)	26 (63.0)	47 (52.8)
Age at onset >70	20 (32.2)	1 (20.0)	8 (50.0)	11 (27)	32 (36.0)
M129 M	60 (96.7)	5 (100)	16 (100)	39 (95.1)	86 (96.6)
M129 V	2 (3.3)	0 (0)	0 (0)	2 (4.9)	3 (3.4)
V129 V	0 (0)	0 (0)	0 (0)	0 (0)	0 (0)
